# Confronting Potential Influenza A (H5N1) Pandemic with Better Vaccines

**DOI:** 10.3201/eid1310.061262

**Published:** 2007-10

**Authors:** Azizul Haque, Didier Hober, Lloyd H. Kasper

**Affiliations:** * Centre National de la Recherche Scientifique, Paris, France; † Dartmouth Medical School, Lebanon, New Hampshire, USA; ‡ Université Lille 2, Lille, France

**Keywords:** Vaccine, influenza (H5N1), virus, pandemic, antibody, CTL response, broad-spectrum immunity, cytokines, therapeutic vaccines, perspective

## Abstract

Influenza A (H5N1) viruses are strong candidates for causing the next influenza pandemic if they acquire the ability for efficient human-to-human transmission. A major public health goal is to make efficacious vaccines against these viruses by using novel approaches, including cell-culture system, reverse genetics, and adjuvant development. Important consideration for the strategy includes preparation of vaccines from a currently circulating strain to induce broad-spectrum immunity toward newly emerged human H5 strains. This strategy would be a good solution early in a pandemic until an antigenically matched and approved vaccine is produced. The concept of therapeutic vaccines (e.g., antidisease vaccine) directed at diminishing the cytokine storm frequently seen in subtype H5N1–infected persons is underscored. Better understanding of host–virus interaction is essential to identify tools to produce effective vaccines against influenza (H5N1).

## Abstract 2

Better understanding of host-virus interaction is essential to produce effective vaccines against influenza (H5N1) viruses.

Bird flu caused by the influenza A virus subtype H5N1 has spread with alarming speed across Europe, Africa, and parts of Asia in which the infection was not reported earlier. Establishment of the highly pathogenic avian influenza (H5N1) as an endemic virus within duck and poultry populations and its capacity to cross species barriers increase the possibility of adaptation to humans and a pandemic. Human influenza infections with subtype H5N1 viruses are often fatal. As of June 4, 2007, 309 laboratory-confirmed cases of human infection have been reported to the World Health Organization (WHO); 61% were fatal, mainly in persons 10–39 years of age (www.who.int/csr/caculatordisease/avian_influenza/en). If a pandemic is triggered by transmissibility of influenza (H5N1) from person to person, millions of people could die, and economies would likely be crippled for 6–24 months.

In the event of a pandemic, vaccination against influenza (H5N1) could limit the impact of infection at a public health level. However, no evidence exists that available vaccines would be protective against the pandemic strain of the virus. We comment on some of the limitations of currently available vaccines and propose novel strategies to improve vaccine formulations against influenza (H5N1).

## Host’s Immune Responses to Influenza (H5N1)

The host response to influenza (H5N1) infection has not been defined, which has proven a considerable challenge in epidemiology and public health research. To develop efficient vaccines, understanding how the virus interacts with the host in natural infection is necessary. Having insights into the hosts’ responses to influenza (H5N1) would help define targets for therapeutic intervention. Whether humans can develop immunity during a primary infection that would control replication and spread of subtype H5N1 viruses has been questioned (*1*). However, marked inflammatory responses develop after infection with influenza (H5N1) in humans and other animals (*2*–*4*). This condition is associated with statistically significant synthesis of various proinflammatory cytokines, such as tumor necrosis factor-α, interleukin (IL)–6, interferon (IFN)-γ, IL-1α, and chemokines, including IP-10, MIG, monocyte chemoattractant protein–1 (MCP-1, IL-8, and RANTES. i.e., regulated on activation, normal T-cell expressed and secreted). If this is the case, these observations are consistent with the possible induction of innate immune responsiveness in the persons infected with influenza (H5N1). Most cases of influenza (H5N1) infection in humans have been described as clinical. However, whether subclinical or asymptomatic infections can develop in some persons is not known. Disease in humans caused by influenza (H5N1) appeared to be milder in Turkey than in eastern Asia (*5*). The death rate of ≈25% was half that of previously known outbreaks, and 5 mild or completely asymptomatic cases have been reported. One theory holds that milder cases have been occurring elsewhere but are not being recorded. Recently, 3 persons among 120 apparently healthy volunteers from the People’s Republic of China, showed detectable virus-neutralizing antibody response to subtype H5N1 before vaccination (*6*). Moreover, pigs infected with subtype H5N1 have become asymptomatic in Indonesia. Are these signs of development of some degree of immunity to virus, containing its replication and thus causing milder infection in naturally infected mammals?

Recently, clusters of bird flu cases were reported in Western Java, Indonesia (*7*); fatal disease developed in 6 persons there from the same family. Two other family members became ill but survived. All the family members likely had similar levels of exposure because they all lived in the same household. Other cases of nonfatal infections have been seen in Thailand and Vietnam. Unfortunately, there is little information about the immune response to the virus in those who survived, which would be valuable for understanding the mechanisms of protection. Indeed, following up the persons (cohort) living in the same affected villages, presumably mostly not exposed to virus, should clarify whether the maintained response reflects boosting through natural exposure. Persons with prior exposure, as measured by antibody or viral RNA at recruitment, would likely have substantially higher responses to the vaccine than those naïve at recruitment if the vaccinating antigen contains homologous or cross-reacting determinants. Conceivably, boosting the “natural” immunity is a desirable outcome to improve protective efficacy of any vaccine approach. Additional studies are required to evaluate the merits of priming populations in advance of an influenza (H5N1) pandemic.

After initial hesitation about using a wide-scale program of poultry vaccination, some European and Asian countries have begun vaccination. Inactivated vaccines are widely used in poultry but lack of critical potency testing, standardization, and quality control has led to variable and suboptimal immune responses. Moreover, a legitimate concern remains that the fowl vaccinated by attenuated live viruses may survive the disease but still carry the virus; thus, they would continue to spread influenza (H5N1) silently at the flock level (*8*) or to humans who come into contact with them. Vaccination that resulted in low levels of seroconversion facilitated the emergence of the Fujian-like sublineage of influenza (H5N1) in poultry (*9*).

The immune responses elicited by subpotent vaccines may exert selection pressure that favors antigenic drift and shift (Figure). Antigenic drift relies on the accumulation of mutations within the antibody-binding sites in the hemagglutinin (HA), neuraminidase (NA), or both that abrogate the binding of antibodies. This makes influenza A virus strains able to evade neutralizing antibody from prior infection or vaccination. Antigenic shift, which is seen only with influenza A viruses, is a more drastic change. It results from genetic shift by reassortment exchange of the HA, and sometimes the NA, with novel subtypes that have not been present in human viruses for a long time. Antigenic shift leads to replacement of circulating strains with new variants that are able to reinfect hosts immune to earlier types; the result is usually a pandemic. Antigenic shifts caused 2 of the major influenza A pandemics in the last century, including the 1957 subtype H2N2 and 1968 subtype H3N2 outbreaks (*10*).

**Figure:**
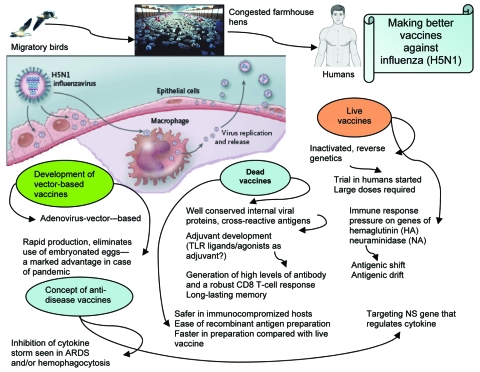
Spreading mode of influenza A (H5N1) viruses and efforts to make better vaccines for potential pandemic. ARDS, acute respiratory distress syndrome.

## Live Vaccines for Use in Humans

Most influenza vaccines used in the United States and Europe are produced in embryonated hens’ eggs and are formaldehyde-inactivated preparations (*11*). Because highly pathogenic influenza (H5N1) subtypes may kill embryonated eggs, use of viruses that are no longer pathogenic, such as H5 (which lacks the polybasic cleavage site), to reduce the virulence of influenza (H5N1) vaccine strains so that these can be efficiently propagated in eggs for vaccine production is feasible (*10*). Virus particles that lack the gene for the nuclear export protein or are defective for the matrix (M2) gene were used as live vaccines in animal models (*12*,*13*); however, whether these replication-defective vaccines will work in humans is not known. Live attenuated (cold-adapted) influenza vaccines have long been used in Russia, and a similar product has been approved for use in the United States (*14*). These vaccines will replicate in the host, and thus lower doses may be effective; however, the preexisting antibody to the virus is more likely to diminish the value of a live vaccine. Moreover, such live vaccines are reported to cause asthma-like reactivity in children (*15*). Monitoring live influenza vaccines is important because the risk for reversion to pathogenicity remains.

With the use of a technique known as reverse genetics, a prototype of influenza virus (H5N1) has been produced for the development of an inactivated subvirion vaccine. The gene segments encoding HA and NA were derived from A/Vietnam/2004, and all other genes were derived from the backbone (A/PR/8/34) virus, commonly used as a platform for influenza vaccine production. The HA gene was further modified to replace the stretch of 6 basic amino acids at the cleavage site, and the resulting virus was avirulent in chickens. In a recent trial, healthy adult volunteers were given 2 intramuscular doses of this inactivated influenza (H5N1) vaccine. This split vaccine induced an antibody response predictive of protection in 54% of healthy adults tested, but only when given intramuscularly at high doses (two 90-μg shots) (*16*). The large amounts needed (2 doses of vaccine, each 6 times the dosage of that used in a standard influenza shot) means that hundreds of millions of doses are needed to tackle a pandemic. Dose-sparing approaches, including the use of an efficient nontoxic adjuvant to boost persons’ immune responses, may improve the vaccine. Another trial was performed with 300 healthy participants 18–40 years of age, in which aluminum hydroxide adjuvant was used with similar split-virus vaccine (*17*). However, the alum-adjuvanted vaccines did not improve the immunogenicity or percentage of seroconversion at lower vaccine doses and only slightly improved immunogenicity at the 30-μg dose. This difficulty underscores the importance of vigorous fundamental research to address the question of how to increase the immunogenicity of such vaccines, whether by better antigen presentation or by choosing alternative routes of administration, so that lesser amount of antigen could be given to induce protective response. The present annual global production capacity is ≈300 million doses of trivalent vaccine containing 15 μg HA per strain. This is equivalent to 900 million doses of monovalent vaccine, a quantity markedly insufficient for the world’s 6.5 billion people. Clearly, dose-sparing formulations are urgently needed.

## Inactivated Vaccines for Immunizing Humans

To test the hypothesis that whole-virion would be more immunogenic than conventional split-virion or subunit vaccines and may be adaptable to the antigen-sparing strategy, an inactivated, monovalent influenza A (H5N1), whole-virion vaccine was prepared from a highly virulent strain A/Vietnam/1194/2004 strain by removing the polybasic amino acids at the cleavage site, making the virus no longer pathogenic. The seed virus was grown to a high titer in embryonated eggs, inactivated with formalin, and purified. These viruses were then adjuvanted with aluminum hydroxide and used in a phase 1 trial (*6*). The highest immune response of 78% seropositivity was observed in the group given 2× 10 μg HA, which is equivalent to that elicited by higher doses of nonadjuvanted (90 μg) or adjuvanted (30 μg) split-virion vaccines (*16*,*17*).

Not knowing which particular genetic variant will sustain human-to-human transmission makes our ability to formulate a vaccine in advance all the more difficult. An inactivated vaccine that induces not only high levels of neutralizing antibody to surface proteins but also CD8 T-cell response against well-conserved antigens derived from internal viral proteins might provide superior protection in an epidemic or pandemic. In cases of established intracellular influenza A infection, infected cells are mainly eliminated by effector CD8+ T cells (CTLs) (*18*). Any vaccine that will induce and direct these CTLs to the site of infection and generate a long-lasting memory response will be more effective for mounting protection against a pandemic form of influenza (H5N1). Inactivated vaccines need to be presented to the host’s immune system with an appropriate adjuvant, but inactivated vaccines that use an adjuvant currently approved for human use (alum or MF-59) usually have lower immunogenicity than live attenuated vaccines (*10*). Therefore, the pursuit for other nontoxic adjuvants, including TLR ligands and agonists that could effectively activate dendritic cells for the presentation of viral antigens to CD4 and CD8 T cells, should vigorously be continued. Use of cytokines such as IL-12 or IL-18 may enhance the immunogenicity of antiviral vaccines. Recombinant fowlpox vaccines coexpressing HA of subtype H5N1 and chicken IL-18 have been shown to induce complete protection in vaccinated chickens (*19*). Use of adjuvants may enhance broader cross-reactive immune responses among influenza viruses (*20*).

## Vaccines that Generate Broad-spectrum Immunity

The evolution of many sublineages of influenza (H5N1) with antigenic diversity in Southeast Asia and southern China favors the wisdom of developing broadly cross-reactive vaccines for protection against an epidemic or pandemic (*21*). Genetically engineered viruses could be constructed; these would express several variant antigens or determinants, thereby generating a broader immune response. The goal would be to develop vaccines that would induce broad-spectrum immunity-conferring protection to influenza including subtype H5N1. Ferrets vaccinated with A/PR/8/34 single-gene reassortants that differed only in their H5s were protected against a lethal challenge with A/Vietnam/1203/04 virus, suggesting generation of cross-protection (*22*). Vaccination of mice with a live attenuated influenza vaccine or an alum-adjuvanted inactivated influenza vaccine based on a related H5 HA from a nonpathogenic avian influenza virus, A/Duck/Pottsdam/1042–6/86 (H5N2), limited the disease severity and reduced deaths following challenge with a current highly pathogenic influenza (H5N1) (*23*). Such cross-protective vaccines may provide clinical protection and prevent deaths in the early stages of a pandemic.

Genes of highly conserved proteins such as the nucleoprotein or M2 proteins could be included in adenovirus vector–based vaccines because immune responses against these influenza viral antigens provide protection in animal models (*24*,*25*). Recently, human adenoviral vector–based HA subtype 5 influenza vaccine induced protection in mice against influenza (H5N1) viruses isolated from humans (*26*,*27*). However, pre-existing immune response to human adenoviruses could be a potential problem in the generation of immune response against a foreign gene of interest. Delivering the vaccine nasally could largely overcome this problem because there appears to be no pre-existing immunity in the upper airways. Moreover, a robust CD8 T-cell response would likely be flexible and able to fight influenza (H5N1).

Ideally, we need an effective vaccine for persons of all ages. However, if the vaccine is in short supply, priming first those persons at high risk (e.g., young children, persons >50 years of age, healthcare workers) may be justifiable. During the early stages of an emerging H5 pandemic, such persons at high risk might be given an adjuvanted vaccine produced from a currently circulating strain, even if it is antigenically distinct, until an optimally matched and approved vaccine is available. This strategy is to produce a vaccine from an antigenically distant influenza (H5N1) strain that could induce broad-spectrum immunity capable of neutralizing newly emerged human H5 strains.

### Cell Culture–based Vaccines

Vaccine development based on a cell culture system has advantages over egg-based technology because H5 strains are highly pathogenic for chickens and supplying large numbers of embryonated eggs could be difficult in a pandemic. In addition, potential allergic reactions to egg components would be avoided by growing the vaccine virus in tissue culture cells. Recently, mammalian cell culture was used for propagating viruses to prepare killed influenza vaccine (*28*). Inactivated influenza vaccines produced with Madin-Darby canine kidney (MDCK) and Vero cells, which served as vaccine substrates, have been licensed in the Netherlands. Of note, the human cell line PER.C6 may provide a useful cell-based system because, unlike MDCK and Vero cell systems, it does not require a solid matrix support for the growth of cells. Selecting background viruses that grow well in these cell cultures and monitoring them for antigenic changes and contaminating microbes during propagation of the virus in cell culture need to be considered.

## Development of “Universal” Vaccines

For the development of a universal influenza vaccine, a possible target is the relatively conserved M2 homotetramer. The concept is based on identifying alternative influenza antigens that are not as susceptible to antigenic shift and drift. Some degree of protection was induced in mice by priming with an M2 ectodomain peptide in adjuvant (*29*). Studies that used the M2eA peptide conjugated to keyhole limpet hemocyanin and *Neisseria meningitidis* outer membrane protein illustrated good immune responses not only in mice but also in ferrets and rhesus monkeys (*30*). In a recent study, 3 M2eA sequences, representing a range of epidemic strains and the (H5N1) strain, were fused to a proprietary hydrophobic protein domain. The resulting fusion proteins, formulated in liposomes, stimulated a protective response in mice challenged with subtypes H1N1, H5N1, H6N2, or H9N2 (*31*). Previous studies have shown that when M2e is linked to hepatitis B virus core (HBc) particles, it becomes highly immunogenic, eliciting protective antibody response in mice (*25*). Recently, a series of M2e–HBc constructs were made by increasing the copy number of M2e inserted at the N terminus from 1 to 3 per monomer. The best protection was seen when mice were vaccinated intranasally with these constructs combined with CTA1-DD, a cholera toxin A1–derived mucosal adjuvant (*32*).

M2 serves as a pH-induced proton channel on the surface of all influenza A viruses but is present in low quantities. Further studies are warranted for understanding the mechanism of immune response to M2eA and for defining the appropriate immunization conditions for humans.

## Vaccination and Correlates of Immune Protection

The lack of established correlates of immunity in animals and humans poses challenges to developing consistent immunologic endpoints for clinical trials and appropriate criteria for vaccine efficacy. Serum antibody titers, mainly those determined by hemagglutination inhibition (HI) or virus neutralization (VN) assays, or both, are considered surrogate measures of protection. However, the HI test is insensitive for the detection of antibody to avian HA; there also are no recognized clinical correlates of immune protection for neutralization antibody (*33*,*34*). Recently, HI or VN assay failed to detect antibodies in ferrets protected by vaccination with whole-virus vaccines containing internal protein from Dk/Sing virus against a heterotypic virus (*34*). Whether the cross-protection reported is mediated by T-cell response is not known.

In recent years, attempts were made to improve the sensitivity of the HI test. More sensitive detection of antibody to avian HA was seen when horse erythrocytes were used in place of turkey erythrocytes in the HI test because influenza virus was better able to bind to a2,3Gal-specific receptor sites on these erythrocytes (*35*). The presence of asparagines at aa223 (H5 numbering) in H5 HA leads to improved sensitivity of the HI test (*22*).

Often the immunogenicity of H5 vaccine candidates is assessed by HI or VN assays, but the basis of protection remains unclear. Nevertheless, the tests that are used to evaluate efficacy of candidate vaccines are based on the assumption that antibody would mediate the protection against infection induced by vaccination, although this has yet to be critically established.

On the basis of initial evidence, inflammation has been proposed as a possible cause or driving force of avian influenza (H5N1). However, components of the inflammatory response might even be beneficial. To address these possibilities, we need to determine whether inflammation in avian influenza is an early event and a manifestation of innate immune response. If it is, some of the mediators of innate immune response, such as cytokine/chemokine levels, can be included in the evaluation of the potency of candidate vaccines. Further humoral response as a correlate for protection can be fine-tuned by determining the titer and isotype of antibody after vaccination. Several issues concerning vaccine efficacy are unresolved: What are the consequences of vaccination for existing influenza (H5N1) infection, the extent of serologic cross-reactivity between the most closely related types of the virus, and the role in clinical protection? Vaccine administration may provide some therapeutic effects for infected persons who have not yet made an immune response but provide none for those with persistent infection associated with measurable humoral immunity.

Clearly, more studies are warranted to establish a highly reproducible assay to measure immunogenicity of a candidate vaccine and to determine adequate correlates of immune protection. Safety and immunogenicity of adjuvanted vaccines or new formulations should be critically assessed, and any fast-track approval of marketing vaccines must not compromise safety.

## Development of Therapeutic (Antidisease) Vaccines

The marked virulence of the 1997 outbreak suggests that influenza A (H5N1) infection may have novel pathogenic mechanisms not seen in human influenza strains. To attempt to understand pathogenicity of this virus, an influenza virus bearing all 8 gene segments of the 1918 pandemic virus, which claimed at least 20–40 million lives, was recently generated in cultured cells. The reconstructed 1918 influenza viruses displayed accelerated activation of host immune response in mice with high levels of chemokines and cytokines in the lungs, resulting in infiltration of inflammatory cells and extensive damage to the lungs with severe hemorrhaging (*36*). The pathogenicity induced by the reconstructed virus showed marked similarity to that reported with influenza (H5N1).

Increasing evidence from mouse models and humans suggests that certain inflammatory mediators are potent drivers of the disease. If this is true, this could have important implications for developing new therapeutics. Acute respiratory distress syndrome, hemophagocytosis, or both, develop in a substantial fraction of patients with influenza (H5N1) infection; both of these conditions are thought to be promoted by overproduction of proinflammatory cytokines (known as a “cytokine storm”) (*37*). Consistent with these observations, cytokine release was markedly enhanced in human macrophages after infection with influenza (H5N1) (*38*). Further, marked enhancement of chemokine and cytokine levels was observed in influenza (H5N1)–infected persons, particularly in those who died, and these correlated with high and disseminated viral replication (*4*). Additionally, influenza (H5N1) viruses appear relatively resistant to the inhibitory effects of host antiviral cytokines, such as interferons (IFNs) (*39*). Thus, the severity of human influenza (H5N1) infection may be related to the induction of excessive proinflammatory responses that can accompany a primary infection and high viral shedding. Increased inflammation was associated with viral replication in the respiratory and extrarespiratory organs of cats experimentally infected with influenza (H5N1) (*3*). Mice infected with the highly pathogenic influenza (H5N1) strain A/HK/156/97, originally obtained from diseased chickens and an ill child in Hong Kong, China (HK), showed reduced ability to activate transforming growth factor–β (TGF-β), a potent anti-inflammatory cytokine, compared to mice infected with less virulent A/Env/HK437/99 viruses (*2*). The reduced ability to activate TGF-β may produce greater inflammation at the site of infection and thus cause more severe disease. Alternatively, the low levels of activated TGF-β in the sera of A/HK/156/97-infected mice may allow the viruses to replicate and spread unchecked in the respiratory tracts of the mice, causing more severe disease. Recently, the impact of the nonstructural (NS) gene variation of Hong Kong (H5N1)/97 on cytokine production was illustrated (*40*). The NS gene reassortant induced elevated pulmonary concentrations of the inflammatory cytokines IL-1α, IL-1β, IL-6, IFN-γ, and chemokine KC and decreased concentrations of the anti-inflammatory cytokine IL-10. This cytokine imbalance is reminiscent of the clinical findings in humans infected with influenza (H5N1)/97 virus and may explain the unusual severity of the disease.

The ability to site specific engineering changes in the virus genome allows us to consider a novel vaccine approach. By engineering a virus with site-specific changes in the genome (for example in NS gene), we may produce influenza virus vaccine that favors the production of beneficial anti-inflammatory cytokines but remains highly immunogenic. In another approach, a human replication-incompetent, adenoviral vector–based influenza vaccine could be developed, in which genes of anti-inflammatory cytokines are coexpressed, which will inhibit overproduction of proinflammatory cytokines. Such vaccines would be considered therapeutic vaccines (e.g. antidisease vaccines), which would inhibit inflammation at the site of infection and protect against severe disease (Figure). Excessive production of anti-inflammatory cytokines may result in an inappropriate suppression of the host immune response. Further studies will validate the beneficial effect of the anti-inflammatory response for temporizing the cytokine storm seen in influenza (H5N1). Development of an immunization protocol that uses an adjuvant that allows selective priming of an antigen-specific immunoregulatory cytokines (e.g., IL-10, TGF-β) would be a major advance in the development of a vaccine for bird flu with a substantial inflammatory component. The search for potential adjuvants, such as TLR ligands and agonists that will favor the synthesis of inhibitory cytokines including IL-10, should be pursued. By testing whether manipulation of inflammatory pathways changes the pathologic course, we would identify new targets for disease intervention.

## Conclusions

Vaccination is the best option by which to prevent the spread of a pandemic virus and reduce the severity of disease. Defining the host response to influenza (H5N1) in natural infection is urgently needed to better understand the basis of protection and subsequent development of efficacious vaccines. Improved vaccine strategies, which will require less antigen and be more robust in inducing both antibody and cell-mediated immunity for neutralizing influenza (H5N1) viruses, should be considered. To create an effective vaccine, a combination of factors must be optimized—such as number of doses, formulation without or with better adjuvant, and dose range. We also need to develop a reproducible assay that measures immunogenicity of a vaccine and to establish adequate correlates of protection. The efficacy of potential cross-reactive vaccine candidates to induce broad-spectrum immunity to influenza (H5N1) viruses should be assessed critically; stockpiling of such vaccines may be justified in the absence of optimally matched and approved vaccine during early stages of an H5 pandemic. Search for therapeutic vaccines (antidisease vaccines) aimed at controlling innate immune responses should be pursued, given the clinical evidence that the H5N1 subtype elicits a cytokine storm that contributes to disease pathogenesis. Vaccine development and deployment need to be undertaken by a partnership of academia, government, and industry. The risk for dissemination of pandemic virus will remain if the disease is controlled in 1 area but not in others. A global approach is vital for combating the next influenza pandemic, a monumental public health challenge.
